# An inflammation-related gene landscape predicts prognosis and response to immunotherapy in virus-associated hepatocellular carcinoma

**DOI:** 10.3389/fonc.2023.1118152

**Published:** 2023-03-09

**Authors:** Ying-jie Gao, Shi-rong Li, Yuan Huang

**Affiliations:** ^1^ Department of Biochemistry and Molecular Biology, School of Bioscience and Technology, Chengdu Medical College, Chengdu, Sichuan, China; ^2^ Laboratory of Animal Tumor Models, Frontiers Science Center for Disease-related Molecular Network, State Key Laboratory of Biotherapy and Cancer Center, National Clinical Research Center for Geriatrics, West China Hospital, Sichuan University, Chengdu, Sichuan, China

**Keywords:** hepatocellular carcinoma, virus, inflammation, tumor microenvironment, immune, drug sensitivity

## Abstract

**Background:**

Due to the viral infection, chronic inflammation significantly increases the likelihood of hepatocellular carcinoma (HCC) development. Nevertheless, an inflammation-based signature aimed to predict the prognosis and therapeutic effect in virus-related HCC has rarely been established.

**Method:**

Based on the integrated analysis, inflammation-associated genes (IRGs) were systematically assessed. We comprehensively investigated the correlation between inflammation and transcriptional profiles, prognosis, and immune cell infiltration. Then, an inflammation-related risk model (IRM) to predict the overall survival (OS) and response to treatment for virus-related HCC patients was constructed and verified. Also, the potential association between IRGs and tumor microenvironment (TME) was investigated. Ultimately, hub genes were validated in plasma samples and cell lines *via* qRT-PCR. After transfection with shCCL20 combined with overSLC7A2, morphological change of SMMC7721 and huh7 cells was observed. Tumorigenicity model in nude mouse was established.

**Results:**

An inflammatory response-related gene signature model, containing *MEP1A*, *CCL20*, *ADORA2B*, *TNFSF9*, *ICAM4*, and *SLC7A2*, was constructed by conjoint analysis of least absolute shrinkage and selection operator (LASSO) Cox regression and gaussian finite mixture model (GMM). Besides, survival analysis attested that higher IRG scores were positively relevant to worse survival outcomes in virus-related HCC patients, which was testified by external validation cohorts (the ICGC cohort and GSE84337 dataset). Univariate and multivariate Cox regression analyses commonly proved that the IRG was an independent prognostic factor for virus-related HCC patients. Thus, a nomogram with clinical factors and IRG was also constructed to superiorly predict the prognosis of patients. Featured with microsatellite instability-high, mutation burden, and immune activation, lower IRG score verified a superior OS for sufferers. Additionally, IRG score was remarkedly correlated with the cancer stem cell index and drug susceptibility. The measurement of plasma samples further validated that CCL20 upexpression and SLC7A2 downexpression were positively related with virus-related HCC patients, which was in accord with the results in cell lines. Furthermore, CCL20 knockdown combined with SLC7A2 overexpression availably weakened the tumor growth *in vivo*.

**Conclusions:**

Collectively, IRG score, serving as a potential candidate, accurately and stably predicted the prognosis and response to immunotherapy in virus-related HCC patients, which could guide individualized treatment decision-making for the sufferers.

## 1 Introduction

Considering as the third leading cause of cancer death worldwide, hepatocellular carcinoma (HCC) is the fifth most usual cancer (1). During HCC progression and development, a battery of risk factors, such as genetical (i.e., alteration of tumor suppressors and oncogenes) and environmental factors (i.e., viruses), had been indicated to be involved (2). Thus, comprehensive understanding of risk factors could assist researchists and clinicians to make effective therapeutic options in terms of HCC treatment. As we all know, various viruses, involving hepatitis B virus (HBV) and hepatitis C virus (HCV) targeting several cellular and molecular pathways, could contribute to HCC pathogenesis (3). As we all know, chronic HBV and HCV infections account for probably 60-70% of the leading cause for hepatocarcinogenesis worldwide (4). Especially in Africa and Asia, HBV is the single primary risk factor for liver cancer, whereas HCV infection dominates in Japan, northern Europe and USA (5). Thus, Hepatitis B and C viruses are an universal health issue for the reason of causing acute and chronic infections, which can generate liver cirrhosis and even HCC with significant mortality more than 1.3 million deaths per year (6, 7).

Presently for advanced HCC, cure options are finite, among which chemotherapy is one of the most vital treatment patterns (8). Multiple tyrosine kinase inhibitors (mTKIs) such as sorafenib, lenvatinib, cabozantinib, and regorafenib have been used to treat advanced HCC. However, although they show some benefit, it does not significantly alter the course of disease for most patients (9, 10). In addition to standard systemic therapy with mTKIs, recent studies demonstrate the capacity for durable responses from immune checkpoint inhibition in subsets of HCC patients across disease etiologies (11). A majority of HCC derives from the context of chronic inflammation, with a lot of cases relevant with hepatitis virus infections, which are associated with both local and systemic immune deficiency (12). Also, the liver is an immunologic organ to enhance or suppress the immune response to cancer arising within it (13, 14). Therefore, there is an imperative to develop an effective gene signature for risk stratification and guiding clinical treatment, especially involved in targeted therapy and immunotherapy.

Chronic inflammation resulting from viral infection markedly enhances the likelihood of cancer development by activating inflammatory signaling pathways and cytokines, stimulating growth of infected cells and inhibiting apoptosis viruses (15–17). Thus, it attested apparent that inflammation is served as a prime driving force in cancer progression for the close correlation between chronic virus infection and carcinogenesis. When it comes to HCC development, there are approximately 90% of primary liver cancers arising almost exclusively in the setting of inflammation (18, 19). Recently, inflammation inhibition has appeared to be as a conducive therapeutic choice, particularly for tumors where conventional treatment is unavailable (20). Presently, the studies are predominantly concentrated upon figuring out the role of individual inflammation-associated genes on HCC progression and prognosis (21–25). In addition, inflammation-associated genes are often deemed as therapeutic targets for tumors since exploring the relevance between inflammation-associated genes and tumor immune status may conduce to further integration of targeted therapy and immunotherapy (26, 27).

In the present study, we identified IRGs in virus-related HCC and constructed a prognostic signature to accurately predict the clinical outcome of virus-related HCC patients and immunotherapeutic effect by least absolute shrinkage and selection operator (LASSO) regression analyses as well as Gaussian Mixture Model (GMM) based on The Cancer Genome Atlas (TCGA, https://www.cancer.gov/tcga) and Gene Expression Omnibus (GEO) databases. Also, the prognosis and tumor microenvironmental characteristics of diverse subtypes based on IRGs as well as corresponding responses to therapy were analyzed. Furthermore, we evaluated the molecular features, prognostic significance, and infiltrating immune cell intensities of the IRGs clusters. Our findings verified a potential relationship between inflammation, prognosis, TME, and the response to immunotherapy in virus-related HCC.

## 2 Materials and methods

### 2.1 Data acquisition

RNA sequencing data and corresponding clinical information of 179 virus-relevant patients with liver cancer were downloaded from TCGA website (https://portal.gdc.cancer.gov/repository). RNA sequencing data and clinical information of another 260 virus-related HCC samples were obtained from ICGA website (https://dcc.icgc.org/projects/LIRI-JP). Besides, patients from GSE84337 (n=75) in the GEO repository was screened to acquire clinical parameters and normalized gene expression data. Clinical information of virus-related liver cancer patients was shown in **Table S2**. Samples lacking significant clinicopathological or survival information were excluded from further analysis.

### 2.2 Curation of inflammation-related genes

200 inflammatory response-related genes were found in the Molecular Signatures database and listed in the **Supplementary Table 1**. Furthermore, t-distributed Stochastic Neighbor Embedding (t-SNE), a nonparametric and unsupervised algorithm, was employed to sort or condense patients into diverse clusters, based on given signatures or hallmarks by using an R package Seurat (28). According to the OS data, two groups were singled out for comparison to determine the “inflammation^high^” and “inflammation^low^” clusters. The limma algorithm was applied to filtrate DEGs between the above two groups, generating genes with false discovery rate (FDR) adjusted p-value<0.05 and absolute value of log2 (fold change)>1 were regarded as inflammation-related DEGs.

### 2.3 Protein–protein interaction network construction

The STRING database (https://string-db.org/) was used to establish the protein–protein interaction (PPI) network among sufferers with co-expression coefficients >0.4. Also, cytoscape software (version 3.7.2) was exploited to visualize the network. Moreover, the hub genes were screened with the MCC algorithm of the cytoHubba plugin. The correlation between the expression of inflammation-related genes was identified by the “reshape2” R package.

### 2.4 Enrichment analysis

To explore the potential mechanisms and pathways about inflammation-related genes, the Gene Ontology (GO) and Kyoto Encyclopedia of Genes and Genomes (KEGG) functional enrichment analysis were conducted among IRGs using the R packages “clusterProfiler,” “enrichplot,” “ggplot2,” and “org.Hs.eg.db.”

### 2.5 Consensus clustering analysis of IRGs

Based on the expression of inflammation-related genes (IRGs), we classified distinct inflammation-regulated groups through consensus clustering with the k-means method. The number of patterns and corresponding homologous stability were defined by consensus clustering algorithm using the R package ConsensusClusterPlus with 1,000 repetitions (29).

### 2.6 Relationship of molecular patterns with TME in virus-related HCC

The immune infiltration characteristics (the immune and stromal scores) of virus-related HCC, based on the RNA-seq dataset of TCGA database LIHC, were evaluated by ESTIMATE algorithm (30). Then, CIBERSORTx was applied to quantify the percentages of 22 immune cell subtypes of each patient in the TME (31). Also, the correlation between the subsets on PD-1, PD-L1, and CTLA-4 expression was assessed.

### 2.7 Construction and validation of inflammation-related gene score

To define preliminary inflammation-related DEGs that were significantly associated with OS in the training cohort, univariate Cox regression analyses using the R package “survival” were further implemented among favorable and risk DEGs, of which *p*<0.05 were regarded as positive. Also, Least Absolute Shrinkage and Selection Operator (LASSO) regression with 10-fold cross-validation was explored to narrow down the prognosis-related DEGs applying the R package “glmnet” (32). Meanwhile, based on the Gaussian finite mixture model (GMM), classification was conducted with model-based hierarchical agglomerative clustering with the R package “mclust” (33). Afterwards, the clusters made up of DEGs were classified by GMM and logistic regression analysis was utilized to construct combined models to predict the OS status for patients. Besides, to calculate the predictive value of models, receiver operating characteristic (ROC) curves were established by assessing the area under curves (AUCs). Subsequently, the risk scores of patients were estimated according to the expression level of each inflammatory response-related gene and its relevant regression coefficient. The formula was established as follows: risk score = ∑iCoefficient (mRNAi) × Expression(mRNAi). On the basis of the median risk scores, patients were divided into high- and low-risk clusters among training and validation cohorts. The Kaplan-Meier analysis was applied to compare the OS between the high- and low-risk groups. The predictive value of the prognostic model was assessed on account of ROC analysis. The principal component analysis (PCA), acquiring a low-dimensional cluster distribution from high-dimensional gene sets, was utilized for validating the sectionalization results.

### 2.8 Clinical significance and classification analysis of the prognostic IRG score

The correlation between IRG score and clinical factors was explored. Univariate Cox and multivariate Cox regression analysis were firstly implemented to prove whether IRG score was an independent prognostic predictor. Ulteriorly, a grouping analysis was conducted to explore whether the IRG score sustained its predictive reliable in disparate subgroups on the basis of multifarious clinical variables. Furthermore, the infiltrating levels of immune cells and immune checkpoint (ICP) were analyzed between the distinct risk subgroups and the relevance between IRG score and tumor mutation burden (TMB) score, microsatellite instability (MSI) score, and cancer stem cells (CSC) score was examined.

### 2.9 Nomogram and calibration

Nomogram was constructed by the rms R package. Calibration curves and decision curve analysis (DCA) were utilized to quantify the consistency between the predicted and the observed results for 3-, and 5-years survival rates (34).

### 2.10 Gene mutation analysis

On the basis of the cBioPortal database, genetic alteration data was acquired. And the number and quality of mutations between the two IRG clusters were analyzed using the R “Maftools” package (35). Subsequently, the online database TIDE (Tumor Immune Dysfunction and Exclusion, http://TIDE.dfci.harvard.edu/) and immunophenotype score (IPS) were calculated to execute immune checkpoint inhibitor response of each virus-related HCC patient in the two groups to assess the value of the IRG in terms of prognostic immunotherapy response.

### 2.11 Prediction of drug susceptibility

The pRRophetic R package was used to predict the half-maximal inhibitory concentration (IC50) value of cancer drugs in diverse risk subgroups, which represented the availability of a substance in inhibiting a particular biological or biochemical process (36, 37).

### 2.12 Clinical samples

The samples contained 58 blood samples from virus-related HCC patients from West China Guangan Hospital, Sichuan University, between March and November in 2019. The diagnoses of HCC were confirmed by senior pathologist. None of the patients experienced radiotherapy or chemotherapy treatment before samples collection. Also, 50 blood samples from healthy people were considered as the control cluster. Informed consent was obtained from all participants for the use of their blood samples in this study. This project was approved by the Clinical Research Ethics Committee of Chengdu medical college.

### 2.13 Cell culture

The human cell lines (WLR68, LO2, Huh-7, SMMC7721, HepG2, and HCCLM3) were obtained from the School of Bioscience and Technology, Chengdu Medical College (ChengDu, China). All of them were cultured in DMEM (Gibco) medium, which were supplemented with 10% fetal bovine serum (FBS) at 37 °C with 5% CO_2_. In addition, the cells were photographed after treatment with paraformaldehyde.

### 2.14 Samples processing, RNA extraction, and real-time fluorescence qRT-PCR

Approximately 8 ml of whole blood from participants was gathered in EDTA tube. After centrifuged at 1,2000g at 4°C to spin down the blood cells for 10 min, the supernatant was shifted into microcentrifuge tubes. Afterwards, plasma was aliquoted or stored at −80°C. RNA was isolated from 400 μL plasma with the mirVana PARIS kit (Ambion, USA) abided by the manufacturer’s protocol. The PrimeScript™ RT reagent kit (TaKaRa) was further applied for reverse transcriptase reaction. Reverse transcription−quantitative PCR (RTqPCR) were implemented to attest the expression levels of the six hub genes in plasma samples and cell lines. The mRNA expression level of *MEP1A*, *CCL20*, *ADORA2B*, *TNFSF9*, *ICAM4*, and *SLC7A2*, was normalized by GAPDH. Fold differences were calculated for each group with normalized CT values.

### 2.15 Cell transfection

Full-length SLC7A2 cDNA was synthesized and cloned into the pCS-CG vector (Addgene, Cambridge, MA, USA). shRNA sequences specifically against CCL20 (shCCL20) and control-shRNA against luciferase (shCtrl) were expressed from pLKO.1-puro (Addgene, Cambridge, MA, USA). Production of lentiviral particles using HEK-293T cells and subsequent infection of Huh7 and SMMC7721 cells were performed according to the manual instructions.

### 2.16 *In vivo* tumorigenicity

14 male nude mice (5-week-old) were purchased from Beijing Vital River Laboratory Animal Technology Co., Ltd. (Beijing, China). SMMC7721 (1×10^7^) stably transfected with shCtrl or shCCL20/overSLC7A2 was subcutaneously injected into the right gluteal region of each nide mice (n=7). After tumor formed, the tumor volume was calculated every 3 days on the basis of the formula: volume(mm^3^)=width2 (mm^2^) *length (mm)/2. All the mice were euthanized and the formed tumors were weighted after 30 days. The animal experiment was approved by the Animal Care Committee of Chengdu Medical College.

### 2.17 Statistical analysis

All analyses were completed on the strength of R language (Version 4.2.1). Student’s t-test, chi-squared test, or Wilcoxon test was applied to compare the differences between groups. Spearman’s correlation test was performed to evaluate the correlation between variables. *p*-value of <0.05 was deemed as statistically positive.

## 3 Results

### 3.1 Workflow of study

**Figure 1:**
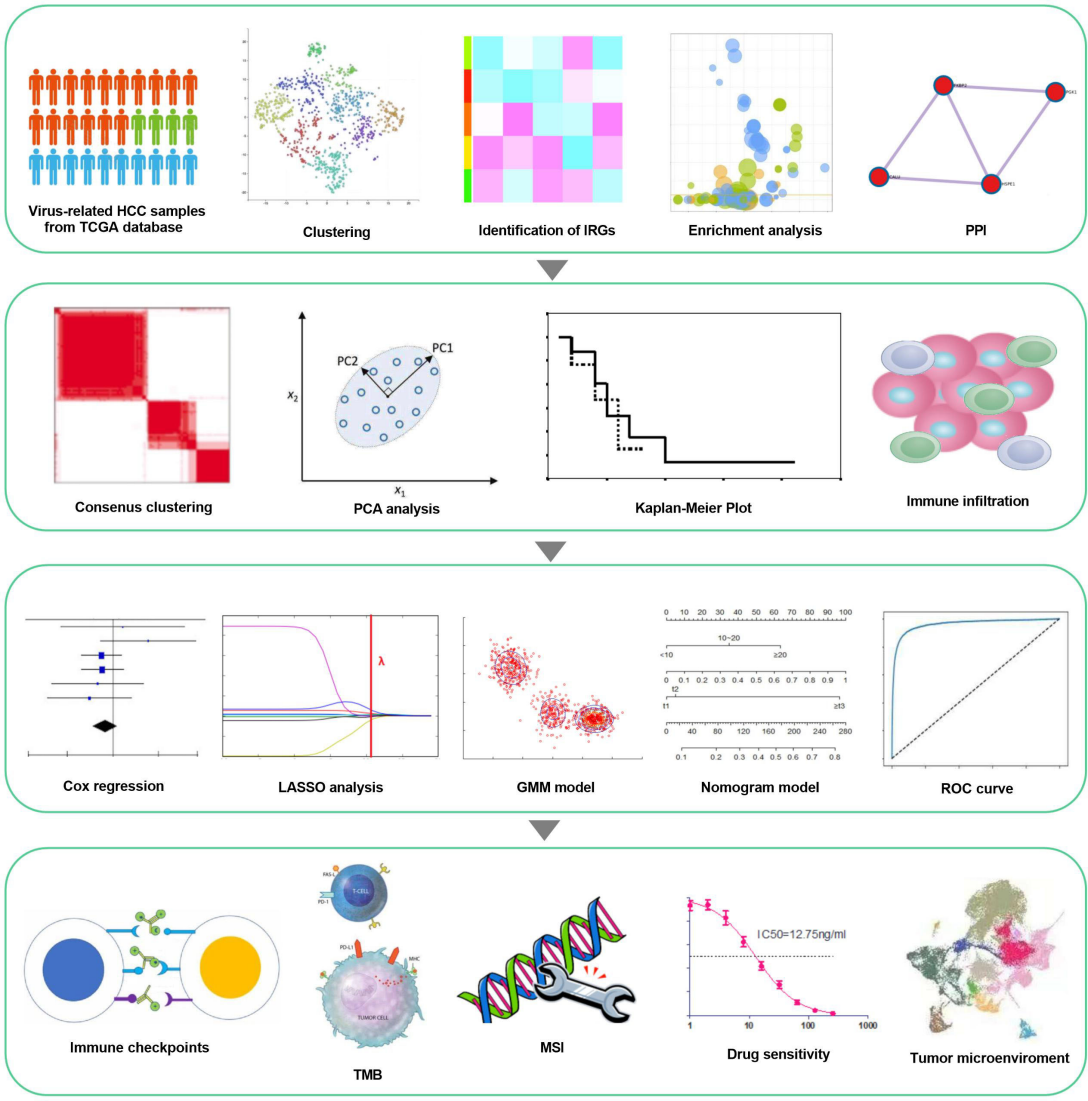
workflow of the study. Virus-related HCCs extracted from TCGA database and 200 inflammation-relevant markers from the Molecular Signatures database were analyzed to identify IRG DEGs. Next, consenus clustering was used to classify inflammation subgroups. The prognostic model was constructed and validated in multiple ways and proved to be stable and reliable. Therefore, based on this model, we also performed analysis about immunological characteristics, drug sensitivity and the correlation between IRGs and Tumor Microenvironment.

The study flowchart is revealed in **Figure 1**, which precise procedure is as follows: First, RNA sequencing from the TCGA database for 179 virus-related HCC sufferers was obtained, as well as 200 IRGs from the Molecular Signatures database. t-SNE, was applied to sort or condense patients into diverse clusters, based on 200 IRGs and IRG DEGs were identified from survival analysis. Next, consenus clustering was classified inflammation subgroups to analyze immune infiltration. Furthermore, a prognostic inflammation-associated model was established, and its corresponding stability was verified with various methods. Ultimately, immunological characteristics and drug sensitivity analysis extended on the idea of clinical application, while the correlation between IRGs and tumor microenvironment in virus-related HCC was attested.

### 3.2 Identification and functional enrichment analysis of inflammation-related differentially expressed genes in virus-associated HCC

**Figure 2:**
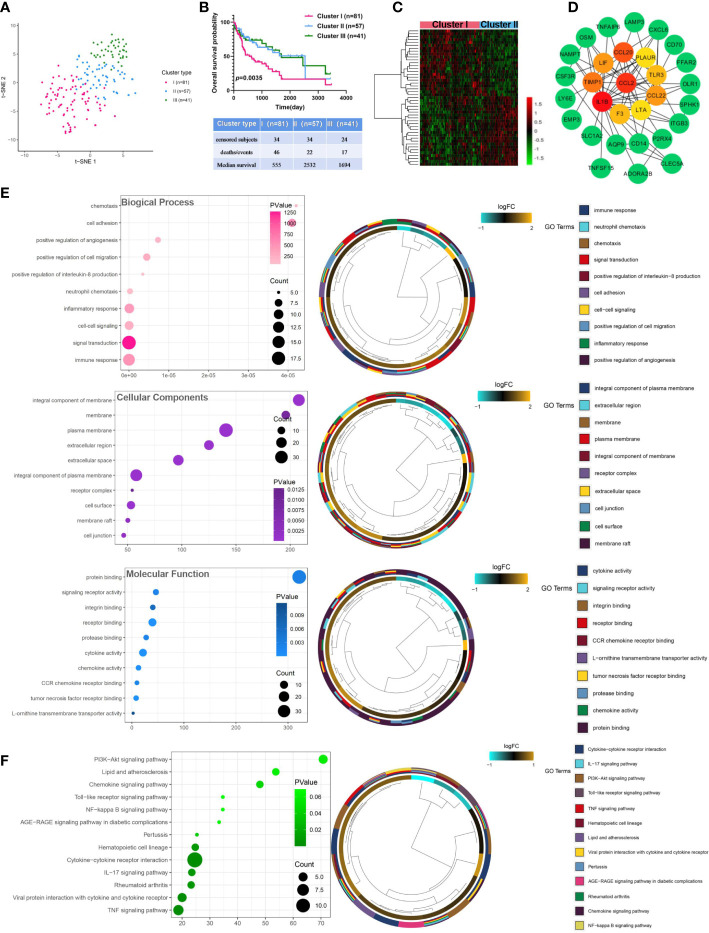
Identification and analysis of inflammation-related differentially expressed genes in virus-related HCC. **(A)** Dot plot for three distinct clusters identified by t-SNE algorithm based on 200 inflammation hallmark genes. **(B)** Kaplan-Meier plot of overall survival for patients in three clusters. **(C)** Heatmap showing expression profiles for inflammation-related DEGs with comparison between cluster I (inflammation^high^) and cluster II (inflammation^low^) groups. **(D)** The Protein-protein interaction (PPI) network between 47 differentially expressed inflammation-related genes (IRGs). **(E)** Gene Ontology (GO) and **(F)** Kyoto Encyclopedia of Genes and Genomes (KEGG) enrichment analysis for IRGs. Adjusted p < 0.01 and p < 0.05 were considered significant.

The expression matrix of 200 IRGs was adopted to compute the euclidean distance between any two patients from 179 virus-related HCCs, and t-SNE algorithm was further condensed the euclidean distance into two-dimensional points. Subsequently, three clusters with virus-related HCC patients were generated and each patient was assigned to its closest (**Figure 2A**), namely 81, 57, and 41 patients in distinct clusters (Cluster I, Cluster II, and Cluster III), respectively. OS analysis displayed that the most significant differences consisted between cluster I and cluster II. Thus, patients in Cluster II yield the best OS while those in Cluster I had the worst prognosis outcome (**Figure 2B**), indicating that Cluster II and Cluster I might represent the lowest and highest status of inflammation. Accordingly, sufferers in Cluster I and Cluster II were classified into “inflammation^high^” and “inflammation^low^” groups, separately. To obtain inflammation-related DEGs, expression profiles were compared between the inflammation^high^ and inflammation^low^ groups, leading to a total of 47 inflammation-related DEGs identified (**Figure 2C**). Next, A PPI network was constructed, composed of 84 nodes. Among all nodes, 10 hub genes, including CCL20, IL1B, CCL2, CCL22, TIMP1, LIF, TLR3, F3, LTA, and PLAUR were distinguished (**Figure 2D**). Further research found that the DEGs were mostly enriched in immune response, integral component of membrane, and signaling receptor activity (**Figure 2E**; **Table S3**). KEGG analysis also demonstrated that these DEGs were closely related to pathways in inflammation, such as IL-17 signaling pathway, TNF signaling pathway, and NF-kappa B signaling pathway (**Figure 2F**; **Table S3**).

### 3.3 Subtypes classification based on inflammation-related gene signatures

**Figure 3:**
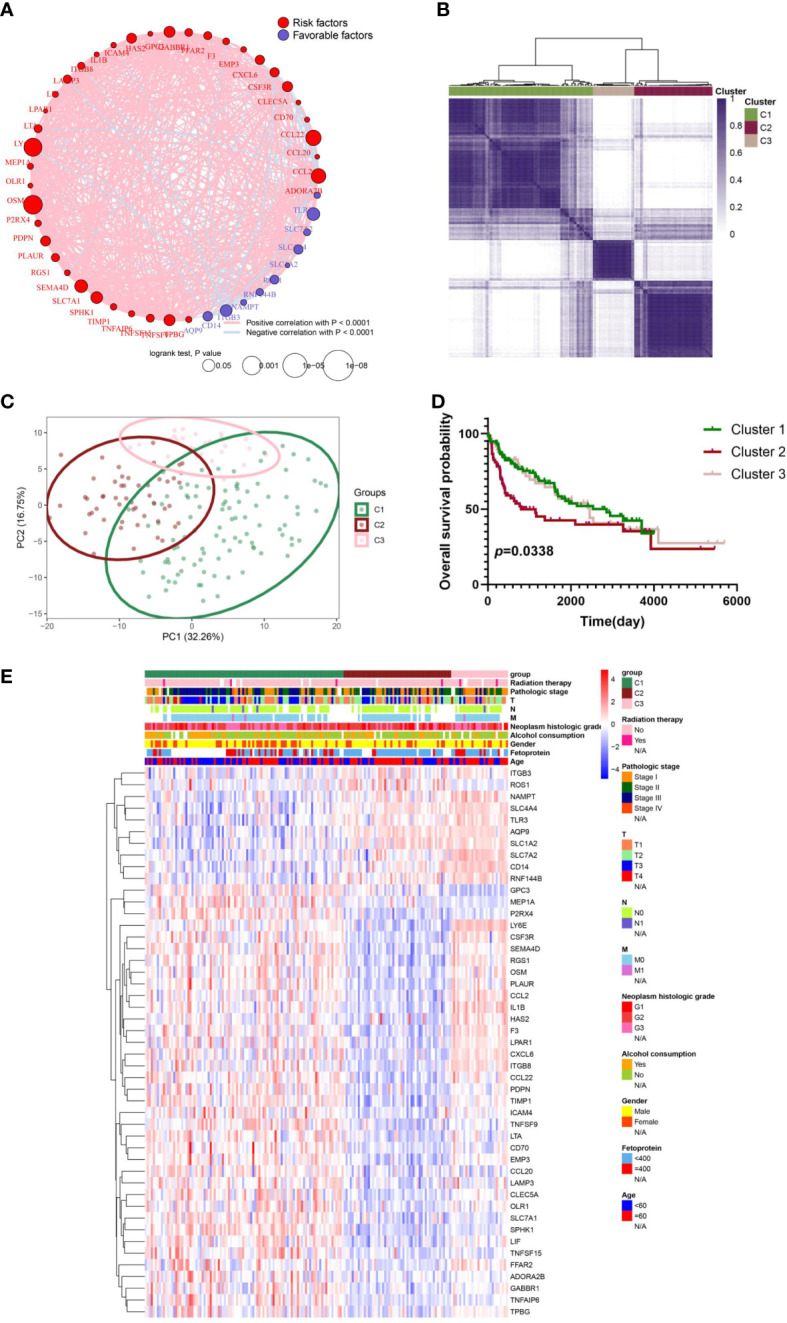
IRG subgroups divided by consistent clustering and its corresponding clinicopathological and biological characteristics. **(A)** The correlation in inflammation-related gene expression. **(B)** Consensus clustering of 179 sufferers from virus-related TCGA-LIHC cohorts based on the IRG DEGs. Consensus matrix for optimal k = 3. **(C)** Principal component analysis (PCA) of TCGA database for optimal k = 3. **(D)** Kaplan-Meier analysis for overall survival (OS) curves of patients in distinct clusters. **(E)** Differences in clinicopathologic characteristics and expression levels of IRGs between the three distinct subgroups.

The relevance network of IRGs interactions, regulator relationships, and corresponding survival status in virus-related HCC patients was presented in **Figure 3A** and **Table S4**. To further conclude the relation between expression profiles of IRGs and HCC subtypes, a consensus clustering analysis was conducted to separate patients into different gene clusters based on the expression levels of the IRGs (**Figure 3B**). Three discrepant patterns were determined: 98 cases in Cluster 1, 53 cases in Cluster 2, and 28 cases in Cluster 3 (**Figure 3C**). Afterwards, OS status of the three patterns was revealed, contributing to a consequential difference observed (**Figure 3D**). Additionally, the genomic expression and clinicopathological features of three clusters were displayed in **Figure 3E**, identifying a substantial difference between IRGs expression and clinical characters.

### 3.4 Discrepancies in TME infiltration for inflammation patterns in virus-related HCCs

**Figure 4:**
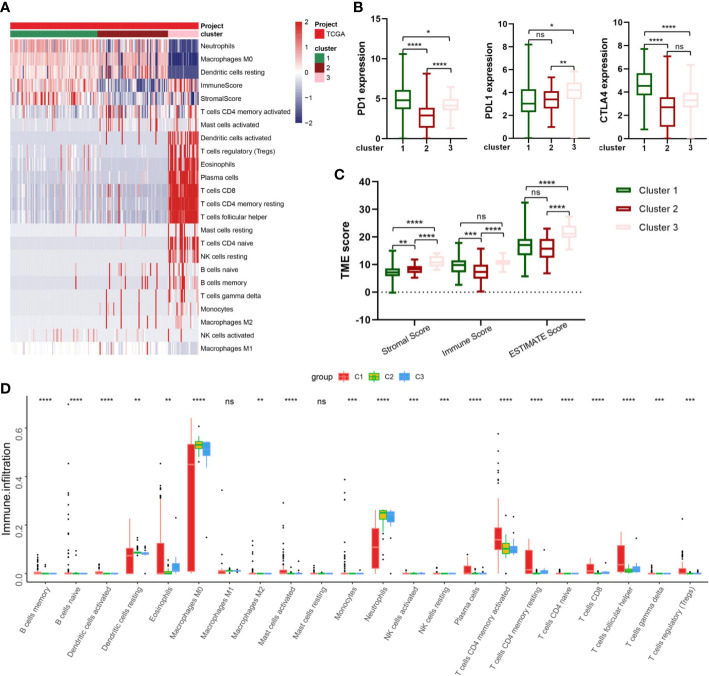
Correlation between IRG subgroups and tumor microenvironment in virus-related liver cancer (TCGA cohort). **(A)** heatmap displaying clustering of tumor-infiltrating immune cells in TCGA cohort. Rows represent tumor-infiltrating immune cells, and columns represent samples. **(B)** Expression levels of PD-1, PD-L1, and CTLA-4 in the three virus-related HCC subgroups. **(C)** Comparison of TME scores among IRG subgroups. **(D)** Abundance of 23 infiltrating immune cell types in the three virus-related HCC subgroups. *p<0.05; **p<0.01; ***p<0.001; ****p<0.0001; ns, not significant.

The CIBERSORT and ESTIMATE algorithms were implemented to confirm the activity or enrichment levels for immune cells in virus-related HCCs (**Table S5**). The heatmap of three independent immune cell infiltration (ICI) subtypes was presented in view of 179 tumor samples with matched ICI profiles from TCGA-LIHC (**Figure 4A**). The expression of three vital ICPs (PD-1, PD-L1, and CTLA-4) was obviously distinct among three clusters. In virtue of the role of TME scores for evaluating the abundance of immune and stromal elements in TME, the ESTIMATE algorithm was further executed to estimate the TME scores, involving stromal score, immune score, and estimate score, in three clusters, the results of which turned out sufferers in cluster 3 yielded superior TME scores (**Figures 4B, C**). In addition, we explored if the three subclasses generated various tumor immune microenvironments (TIME) (**Figure 4D**). Indeed, the immune-high subgroup had high infiltration levels of Eosinophils, Macrophages M0, Macrophages M1, and Neutrophils, while the cluster 1 had remarkable enrichment of resting mast cells.

### 3.5 Development and validation of prognostic IRG score

**Figure 5:**
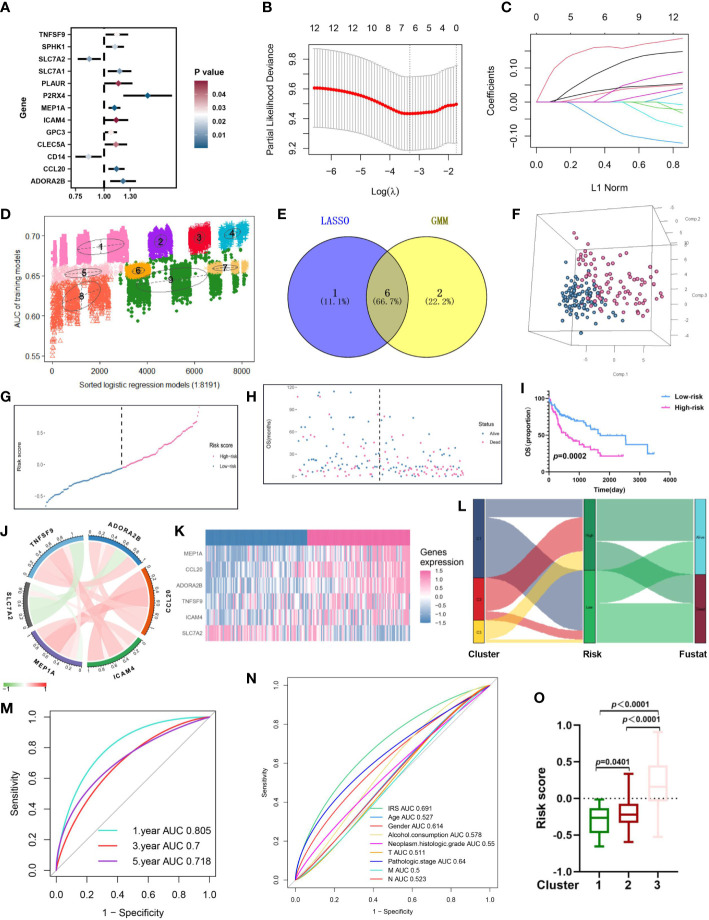
Construction of an inflammation-related risk model to predict the OS of virus-related HCC patients. **(A)** 13 prognosis-related IRGs screened by univariate Cox regression analysis (p<0.05). **(B)** The tuning parameter (λ) in the LASSO model is chosen by the minimum criterion. **(C)** LASSO coefficient distribution of 13 inflammation-related IRGs. **(D)** The pattern of the logistic regression model correlated with the AUC scores and was identified by a Gaussian mixture model. There are nine clusters of 8191 combinations. **(E)** Venn diagram of the shared genes by comparing LASSO model to GMM model. **(F-H)** Principal component analysis, risk score distribution, and survival status distribution for virus-related HCCs from TCGA-LIHC database. **(I)** Kaplan-Meier analysis of the OS between the high group and low group. **(J)** Co-expression network of the hub IRGs. **(K)** Expression patterns of 6 hub prognostic IRGs in high- and low-risk groups. **(L)** Alluvial diagram of subgroup distributions in groups with different IRG scores and clinical outcomes. **(M)** ROC curves for 1 year, 3 years and 5 years. **(N)** ROC analysis showed that the predictive accuracy of IRG was superior to other clinical features. **(O)** Differences in IRG score between the three gene clusters.

Firstly, Univariate Cox regression analysis was utilized on the virus-associated HCC groups, demonstrating that 13 prognosis-related IRGs were correlated with OS (**Figure 5A**; **Table S6**). To prevent model overfitting, LASSO penalized Cox regression modeling and GMM model were simultaneously conducted to filter the vital DEGs, which were positively associated with the prognosis of HCC patients. With the joint method, a novel prognostic gene model with six hub genes (*ADORA2B*, *CCL20*, *ICAM4*, *MEP1A*, *SLC7A2*, and *TNFSF9*) was constructed (**Figures 5B–E**). Then, we computed risk score using the following formula: risk score =∑iCoefficient (mRNAi) × Expression(mRNAi), where i, stands for the expression of six key IRGs. In line with the median risk score, samples were clustered into low- and high-risk subgroups. The distribution patterns from PCA analysis illustrated that patients could be distinguished into high- and low-risk classes (**Figure 5F**). Also, the risk plot of IRG score proved that OS time decreased while mortality rise, as IRG score increased. And survival analysis testified that samples in the low-risk cluster produced significantly longer OS time in comparison with that of the high-risk patients (**Figure 5I**, *P*<0.01, log-rank test) (**Figures 5G–I**; **Figure S1**). Moreover, the expressive relationship among them and heatmap of selected genes were displayed in **Figure 5J** and **Figure 5K**, respectively. To comprehend the relationship between immune subtypes and IRG score, an alluvial diagram was drawn for clusters with distinct risk-subgroups, and accompanying survival status (**Figure 5L**). The outcomes demonstrated that cluster 3 with higher IRG score was most likely associated with death. Whereas the cluster 1 exhibited a lower IRG score and best prognosis status. A time-dependent ROC curve was further performed and the area under the curve (AUC) reached 0.805, 0.7, and 0.718 at 1, 3, and 5 years, respectively (**Figure 5M**). Besides, the ROC curve explained that the predictive OS accuracy of IRG score was superior to other clinical parameters (Age, gender, Alcohol consumption, Neoplasm histologic grade, and TNM stage and age) (**Figure 5N**). Tremendous differences in the IRG score of three clusters were discovered (**Figure 5O**), implying a higher IRG score may be relevant with immune activation-associated features.

### 3.6 Independent prognostic value of IRG score

To explore the relation between the IRG score and clinicopathological Characteristics, the interaction between IRG score and multitudinous clinical parameters (Age, Alcohol consumption, gender, TNM stage, Fetoprotein, Radiation therapy and survival status) was discussed (**Figures S2A–F**). We perceived that IRG scores increased along with the stage III-IV and higher level of fetoprotein. And Univariate and multivariate Cox regression analyses were further conducted to guesstimate the accuracy of the risk model and disclose whether IRG score could be considered as an independent prognostic factor for patients’ prognosis. Accordingly, Univariate Cox regression analysis revealed that both the IRG score and the stage were significantly correlated with OS of the patient (**Table S7**). Furthermore, to uncover the prognostic significance of IRG score in virus-related HCC patients, the patients were assigned into different subgroups based on above clinical parameters. Totally, the high-risk patient’s survival was generally poorer compared to low-risk patients (**Figures S2A–F**).

### 3.7 Establishment of nomogram model

**Figure 6:**
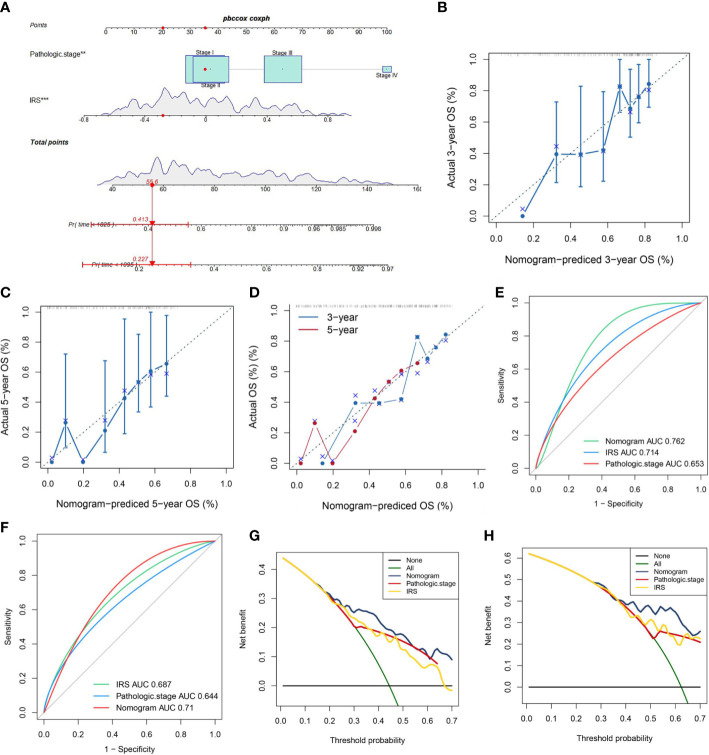
A Nomogram model’ s Construction. **(A)** Nomogram combining pathological stage and risk score predicts 3-, and 5-years overall survival. **(B–D)** Calibration curves test the agreement between actual and predicted results at 1, 3, and 5 years. **(E, F)** Clinicopathological features and the predictive accuracy of the nomograms compared for 3−, and 5−year OS in virus-related HCC, respectively. **(G, H)** The DCA curves of the nomograms at 3−, and 5−year OS in HCC, separately.

As disclosed in **Figure 6A**, a nomogram was reciprocally constructed on the foundation of IRG scores, combined with clinical features. Followly, calibration curves defined the reliability and accuracy of nomogram to predict 3-, and 5-year prognosis (**Figures 6B–D**). As shown in **Figures 6E–H**, the AUC values were as expected, implying this nomogram had an excellent predictive ability for prognosis. Moreover, we also found that this prognostic model with diverse clinical factors presented more net benefits for predicting the prognosis.

### 3.8 Estimation of relation between TME and ICPs in inequable sectionalizations

**Figure 7:**
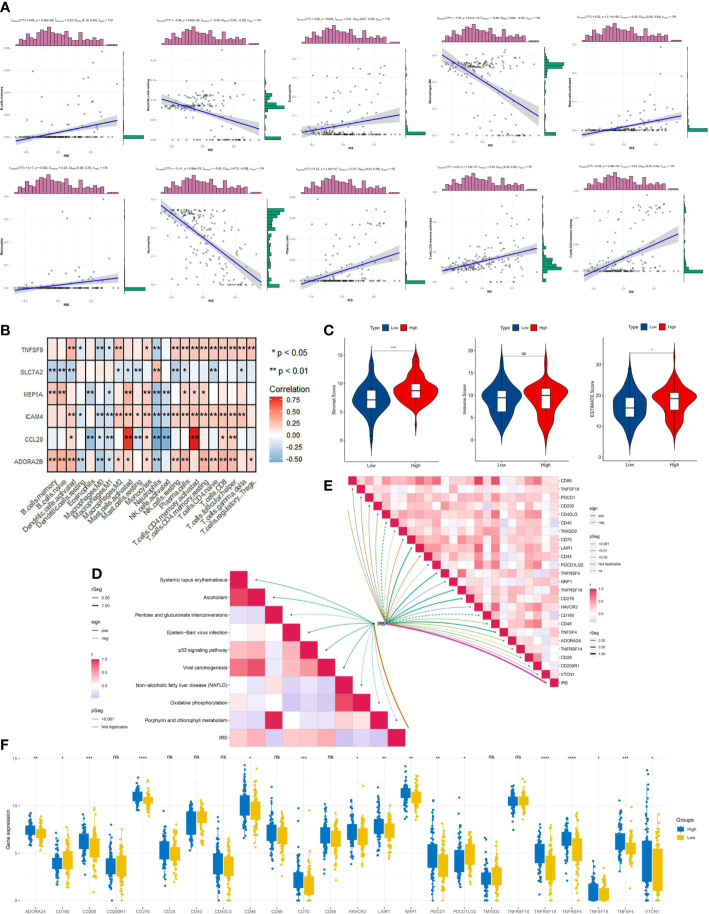
Immune signatures of different risk groups. **(A)** Correlations between IRG and immune cell types. **(B)** The correlations of immune cell infiltration and the hub six genes in the risk model. **(C)** Comparison of immune-related scores between low-risk and high-risk groups. **(D)** the association between IRG and the enrichment scores of immunotherapy response-related gene signatures or **(E)** IRG and the expression of many immune checkpoints. **(F)** The differentially expressed immune checkpoint-related genes between the high- and low risk groups. *p < 0.05, **p < 0.01, ***p < 0.001, ****p < 0.0001, ns, not significant.

We aimed to assess the relevance between IRG score and immune cells abundance with the CIBERSORT algorithm. As depicted in **Figure 7A** and **Figure S3**, the IRG score was significantly associated with the infiltration of B cells memory, Eosinophils, Mast cells activated, Monocytes, Plasma cells, T cells CD4 memory activated, T cells CD4 memory resting, B cells naive, Macrophages M2, Dendritic cells activated, NK cells resting, T cells CD4 naive, T cells gamma delta, T cells CD8, and T cells follicular helper, while the negative performance appeared in relationship with IRG score and Dendritic cells resting, Macrophages M0, and Neutrophils. Then, the correlation between immune cell infiltration and expression status of six genes incorporated with the prognostic model construction was analyzed in **Figure 7B**. Also, high-risk patients experienced higher EstimateScore and StromalScore levels than those in low-risk group (*p*<0.05) (**Figure 7C**). Meanwhile, IRG score was positively associated with the expression of a series of immune checkpoints (such as CD200, CD70, and PDCD1) (**Figure 7E**) and the enrichment scores of immunotherapy response-related gene signatures (**Figure 7D**). Furthermore, we assessed the relationship between ICPs and risk group, demonstrating that ICPs (PD-1, LAIR1, and VTCN1, et al) were inconsistently distributed in two risk clusters (**Figure 7F**).

### 3.9 IRG score-based tumor microenvironment, and stemness analyses

**Figure 8:**
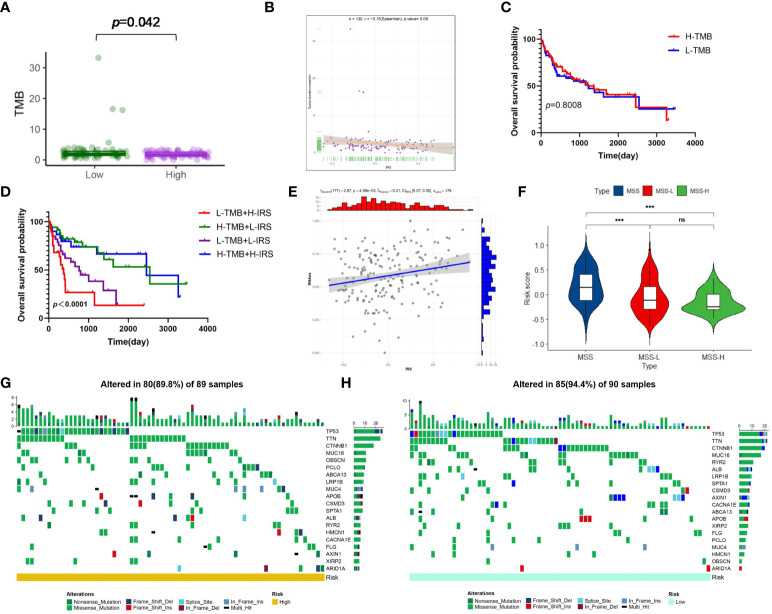
Risk signature-based tumor mutation burden (TMB), microsatellite instability (MSI), stemness analyses, and somatic mutation features. **(A)** The difference in TMB between the high- and low-risk groups. **(B)** Spearman’s correlation analyses between IRG and TMB. **(C)** Kaplan-Meier analysis of the OS between the low- and high-TMB groups. **(D)** The comparison of OS among four subgroups stratified by both TMB and IRG score. **(E)** Correlation between IRG and mRNAsi scores (RNAss). **(F)** Relationships between IRG and MSI. The waterfall plot showing the differences in somatic genomic mutation between **(G)** the high- and **(H)** low-risk groups. *p < 0.05, **p < 0.01, ***p < 0.001, ns, not significant.

Present studies declared that ICP inhibitors were favorable to populations with increased TMB or higher MSI, uncovering that TMB and MSI were both ponderable indexes for predicting tumor immune response (38, 39). As **Figure 8A** demonstrated, that TMB in the low-risk cluster was higher than high-risk cluster suggested that immunotherapy provided more benefits for patients with high risk. And a negative correlation was drawn between IRG score and TMB in spite of the meaningless value (R=-0.15, *p*=0.08, **Figure 8B**). To explore the impact of TMB status on prognosis in virus-related HCC patients, we also conducted survival analysis in various TMB classes. No significant difference of prognosis was revealed between High-TMB patients and low-TMB patients (**Figure 8C**). However, the survival analysis for combination of TMB and IRG score for virus-related HCC patients drew a conclusion that the prognostic benefit in the high-TMB group was eliminated by IRG score (**Figure 8D**). The measurement of RNA stemness score (RNAss) could represent cancer stemness, based on mRNA expression (40, 41). The relevance of IRG score and CSC score was presented in **Figure 8E**. Likewise, lower IRG score was connected with MSI-H pattern, while higher IRG score was linked with the microsatellite stable (MSS) pattern (**Figure 8F**), which also illustrated that low-risk patients may be more susceptive to immunotherapy. Meanwhile, genomic alterations in high and low groups were further analyzed. A rough similarity in the kinds of the top 30 genes with the highest mutation frequency between the low and high groups emerged (**Figures 8G, H**).

### 3.10 Drug sensitivity analysis

**Figure 9:**
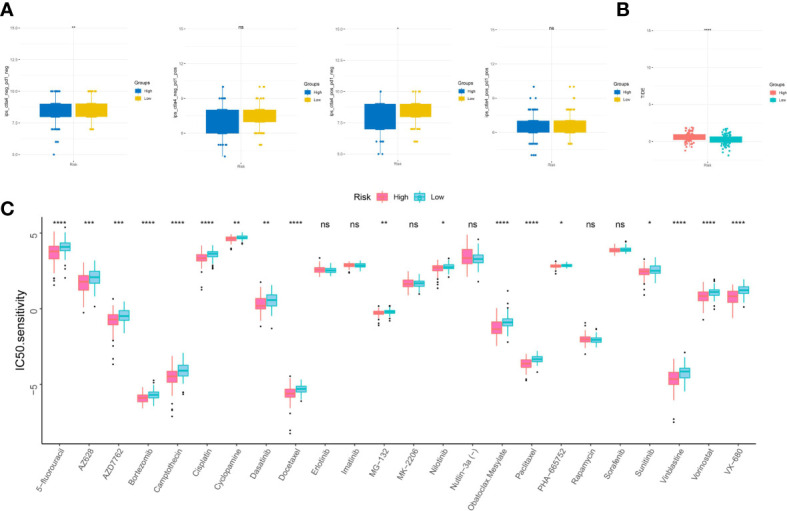
Sensitivity to drugs in virus-related HCC patients with different inflammation-related risk score subgroups. **(A)** immunophenotype score (IPS) and **(B)** tumor immune dysfunction and exclusion (TIDE) in different IRG score groups. **(C)** Relationships between IRG and chemotherapeutic sensitivity. *p < 0.05, **p < 0.01, ***p < 0.001, ****p < 0.0001, ns, not significant.

TIDE scores and IPS scores were conducted to make prediction for sufferers’ responsiveness for appraising the immune response of virus-related HCC patients. Analysis results in **Figures 9A,B** revealed that patients at low-risk generated a lower TIDE score and a higher IPS score, which demonstrated that they may suffer more sensitivity from immunotherapy (42, 43). In the following, aimed at analyzing the clinical application of IRG model, we calculated the alterations in terms of drug sensitivity between diverse risk clusters, reflecting that 5-fluorouracil, AZ628, AZD7762, Bortezomib, Camptothecin, Cisplatin, Cyclopamine, Dasatinib, Docetaxel, MG-132, Nilotinib, Obatoclax Mesylate, Paclitaxel, PHA-665752, Sunitinib, Vinblastine, Vorinostat, and VX-680 yielded advantageous effectiveness for low-risk patients (**Figure 9C**).

### 3.11 Validation of the expression levels of hub genes *in vitro* experiment

**Figure 10:**
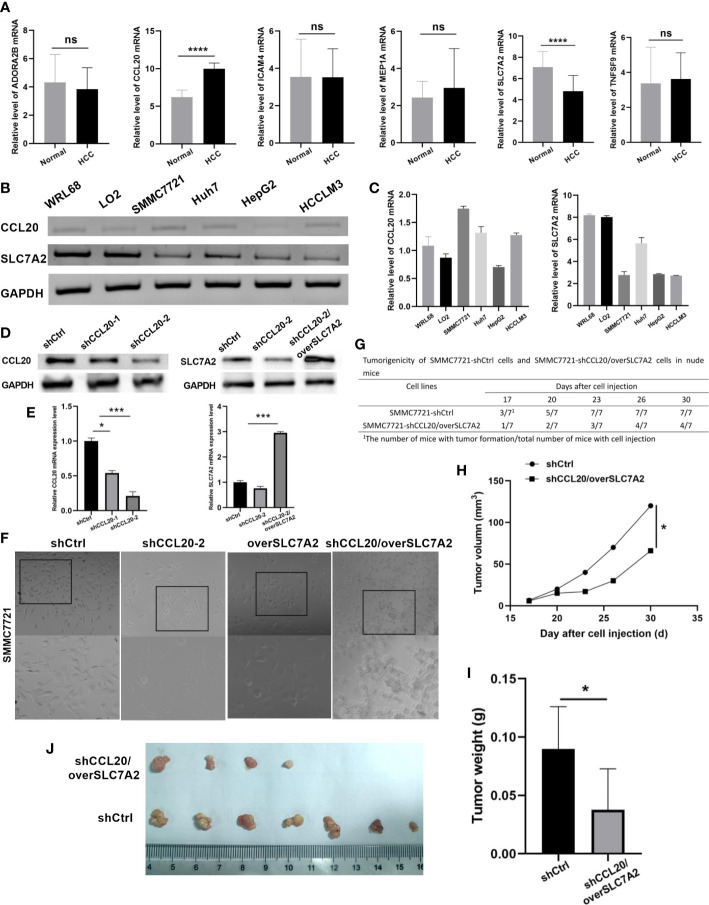
Validation of expression and tumorigenicity of hub genes. **(A)** qRT-PCR validation of MEP1A, CCL20, ADORA2B, TNFSF9, ICAM4, and SLC7A2 in HCC and normal plasmas. **(B, C)** The mRNA expression level of CCL20 and SLC7A2 in HCC cell lines (SMMC7721, Huh7, HepG2, and HCCLM3) and the normal liver cell lines (WLR68, and LO2) was indicated by qRT-PCR assays. **(D)** The protein and **(E)** mRNA expression of CCL20 and SLC7A2 was analyzed by western blotting and RT-PCR in stable SMMC-7721 cells expressing-shRNA against luciferase or CCL20 and over SLC7A2. **(F)** Morphology of HCC cells after knockdown of CCL20 and overexpression of SLC7A2. **(G)** Tumorigenicity of SMMC7721-shCtrl cells and SMMC7721-shCCL20/overSLC7A2 cells in nude mice. **(H)** Tumor volume was measured every 3 days after tumor formation in nude mice injected with SMMC7721 cells transfected with shCtrl or shCCL20/overSLC7A2. **(I, J)** Tumor weight was measured in nude mice injected with SMMC7721 cells transfected with shCtrl or shCCL20/overSLC7A2 after 30 days. *p < 0.05; **p < 0.01; ***p < 0.005; ****p < 0.0001, ns, not significant.

qRT-PCR was applied to verify the mRNA expression levels of six hub genes in plasma samples from 58 virus-related HCC patients and 50 normal people. The unpaired t-test was performed to compute the differences between the virus-related HCC plasma samples and normal plasma samples. And plasma samples validated that the significant differences existed in the expression levels of CCL20, and SLC7A2 between HCC and normal tissues, that is, CCL20 was highly expressed in most HCC plasma samples while the expression of SLC7A2 was significantly higher in normal plasma samples than in virus-related HCC plasma samples (**Figure 10A**). Subsequently, we extracted total RNA from different tumor cell lines (SMMC7721, Huh7, HepG2, and HCCLM3) and the normal liver cell lines (WLR68, and LO2) to measure the mRNA expression levels of CCL20, and SLC7A2. qRT-PCR assays were implemented and the results showed that the mRNA expression levels of CCL20 were significantly higher in liver cancer cells, like SMMC7721, Huh7, HepG2, and HCCLM3, than in normal cells, such as WLR68, and LO2. However, the consequence for SLC7A2 expression level was adverse (**Figures 10B, C**).

### 3.12 CCL20 knockdown combined with SLC7A2 overexpression inhibited tumor growth *in vivo*

To observe the function of CCL20 and SLC7A2 during hepatocarcinogenesis, CCL20 was silenced by transfection with shCCL20 in SMMC7721 cells. In addition, SLC7A2 was further overexpressed based on the SMMC7721 cells with silence of CCL20. First, we successfully constructed two lentiviral vectors harboring shRNA-CCL20-1, and shRNA-CCL20-2, respectively, and established two stable knockdown cell lines in SMMC-7721. The two different shRNAs, especially shRNA-CCL20-2, effectively knocked down the expression of CCL20. Also, SLC7A2 overexpression was indicated *in vitro* (**Figures 10D, E**). Afterwards, we examined the effect of shCCL20, overSLC7A2, and shCCL20/overSLC7A2 on the morphology of SMMC7721 cells. Compared with control cells, shCCL20 cells, and overSLC7A2 cells, showing a spindle-like shape with scattered growth, cells with knockdown of CCL20 combined with SLC7A2 overexpression induced a cobblestone-like appearance with the significant dispersion change (**Figure 10F**). Furthermore, the study established nude mouse tumor xenograft models injected by SMMC7721 cells that were transfected with shCtrl or shCCL20/overSLC7A2. Tumor volume was measured every 3 days (**Figure 10G**). We found that CCL20 knockdown combined with SLC7A2 overexpression significantly lessened the tumor volume (**Figure 10H**). After 30 days, we measured the tumor weight and observed that tumor weight was distinctly lowered by CCL20 knockdown combined with SLC7A2 overexpression (**Figures 10I, J**).

## 4 Discussion

Due to several factors, like vaccination policies and migration, virus infection sustains a health problem publicly and globally with changing epidemiology (44). Presently, virus infection has been documented by an incremental risk of developing chronic HBV infection (CHB), progression to liver fibrosis and end-stage liver disease (ESLD) and evolution of HCC (45). Despite great improvements in the matter of HCC treatment, tumor recidivation triggered by metastasis and drug resistance are still unamiable to HCC sufferers (46, 47). Thus, if we could make early diagnosis and predict the therapeutic effect with a small number of biomarkers, the HCC sufferers would benefit a lot from the risk warning. Previous studies indicated that serum biomarkers, including circulating tumor cells or nucleic acids, and the combination of retinol and retinal panel had preeminent accuracy for HCC prognosis (48, 49). Also, inflammatory response-associated biomarkers in serum, such as medium-granulocyte ratio, platelet-lymphoid ratio and lymphoid-monocyte ratio, have an excellent performance to predict HCC prognosis (50). Accumulative evidence has testified the inevitable relationship between inflammation and intrinsic immunity (51), illustrating that inflammation targeting may serve a vital role to facilitate tumor immunotherapy. However, numerous reports have only emphasized a single inflammatory-related marker or a specific immune cell subtype. Besides, few studies concentrated on the association between inflammation and virus-related HCC. Hence, it is indispensable to clarify the holistic impact and TME infiltration characters regulated by the combinatorial action of disparate IRGs. All the IRGs based on Molecular Signatures Database (MSigDB) were accumulated and several HCC datasets, were applied systematically and comprehensively to filtrate the hub IRG DEGs to establish an inflammation-related model, for probing the distinction of risk models in immune cell infiltration, immune checkpoints, and drug sensitivity to offer clinical prognostic information and guide treatment for virus-related HCC patients.

In this study, 47 inflammation-related signatures were identified and analyzed in TCGA-LIHC database. The candidates were mainly enriched in immune response, IL-17 signaling pathway, TNF signaling pathway, and NF-kappa B signaling pathway. Consistent with other studies (52–55), Chronic inflammation and the presence of inflammatory cells (mainly macrophages) at the tumor site are highly correlated with specific malignancies. Also, cytokines, incorporating tumor necrosis factor (TNF) and Interleukins (IL), can regulate host responses to infection, immune, inflammation, and trauma. Besides, nuclear factor-kappa B (NF-kappa B) comprised of a series of transcription factors regulate the expression of numerous genes included in inflammation and cell proliferation. The results explain that inflammation converts not only inflammatory cells but also alters cytokines to act in collaboration with specific cytokine inhibitors and soluble cytokine receptors to regulate the immune response. Based on these signatures, consensus clustering analysis proved that patients could be divided into three clusters, and there were significant distinctions in the OS among them. The findings revealed that inflammation in virus-related HCC is heterogeneous and sufferers with diverse inflammatory patterns have disparate prognoses.

Subsequently, through the combination of univariable Cox regression analysis, GMM, and LASSO Cox regression analysis, we screened 6 survival-related key signatures, including Meprin A Alpha (MEP1A), CC chemokine ligand 2 (CCL2), Adenosine A2b receptor subtype (ADORA2B), Tumor necrosis factor superfamily member 9 (TNFSF9), Intracellular adhesion molecule 4 (ICAM4), and Solute carrier family 7 member 2 (SLC7A2). They all had been reported to be involved in inflammation or HCC progression previously (56–61). MEP1A, a zinc metalloprotease, was reported to participate in the regulation of inflammatory response and fibrosis. Further analyses verified that MEP1A played a crucial role to regulate cytoskeletal events and accelerated HCC cell proliferation, migration, and invasion (62). Also, In HCC patients, CCL2 was highly expressed and regarded as a prognostic factor. Farther blockade of CCL2/CCR2 signaling restrained liver tumour growth *via* stimulating T cell antitumor immune response (63). ADORA2B functioned as an endogenous feedback loop to dominate hypoxia-relevant inflammation, which was transcriptionally induced under hypoxia or inflammation by hypoxia-inducible transcription factor HIF1A (64). Furthermore, ADORA2B expression was negatively associated with OS of HCC patients. Accordingly, compared with control groups, mice treated with sorafenib in combination with ADORA2B blockage reagents emerged evident inhibition of tumor progression (65). TNFSF9, also known as CD137L and 4-1BBL, had been exhibited in cancer immunotherapy in virtue of the role as a T-cell co-stimulator. Shen YL, et al. considered that TNFSF9 expression was downregulated in roughly 70% of HCC tissues. Thus, TNFSF9 may be a tumor suppressor, deemed as a therapeutic target for HCC (59). As for ICAM4, the studies uncovered that it was vital for immune synapse formation between NK cells and HCC cells to advance NK-mediated immunotherapeutic effects (60). SLC7A2, a member of the solute carrier family, was an independent risk factor for the prognosis of HCC patients if reduced. SLC7A2 Upregulation reduced HCC invasion and metastasis, whereas its downregulation boosted invasion and metastasis. Hence, SLC7A2 may offer novel mechanistic insight into the cancer-promoting property of HCC patients (61). The concrete mechanisms about the signatures in inflammation, immunotherapy, and drug reactivity of virus-related HCC sustained vague, which was one of the limitations of the study. We would continue to study them further in the future.

Based on the six genes, IRG score was calculated to construct a prognostic model for prediction of virus-related HCC patients. IRG score was obviously relevant to clinicopathological features of virus-related HCC. After confounding parameters were controlled, the results attested that IRG score was an independent predictor for virus-related HCC patients’ prognosis. ROCs further showed its prediction robustness for 1-, 3-, and 5-year OS. Thus, IRG score may generate a reliable capacity to make prediction for sufferers’ prognoses. The accumulation of gene mutations leaded to carcinogenesis and was interrelated with inflammation (66). Our findings demonstrated that an apparent difference existed between low and high IRG score in terms of genomic alterations. Huo J, et al. confirmed that preferable prognosis originated from HCC patients with higher TMB (67). Although it is not imperfectly consistent with our findings to some extent, the prognostic benefit in the high-TMB group was eliminated by IRG score after combining TMB and IRG score for survival analysis. These findings further demonstrated the prognostic robustness of IRG score in virus-related HCC patients. Some clinicopathological characteristics, such as TNM stage, was also identified as an independent negative prognostic factor for patients. Therefore, we further constructed a nomogram using IRG score combined with TNM stage to better predict the survival of patients.

Current reports have ascertained crosstalk between cellular metabolic writing and TME remodeling (68, 69). Although numerous HCC patients produced a poor response to immunotherapy, the improvement of immune response efficiency had been the emphasis of immune research (70). In the present study, we quantified tumor inflammation through the calculated IGR score based on the construction of the IRM, objectively displaying the relationship between the inflammation reprogramming and immune microenvironment, aimed at conducting the distinct treatment methods of the two groups. For instance, CD4+, CD8+, B cells, and macrophage cells were infiltrated in the high-IRG subgroup. Also, immune interactions were pivotal characteristics of carcinogenesis and therapeutic target for HCC. In the TME, stromal cells and immune cells were the essential elements, which scores were connected with clinical characteristics and prognosis of HCC sufferers (71). We calculated these scores with the ESTIMATE algorithm and found that a high IRG score cluster significantly showed higher ESTIMATE and stromal scores than a low IRG score cluster. The results suggested that inflammation could be associated with the involvement of TME, thus regulating neoplastic occurrence and development. Therefore, to make quantification of tumor inflammation *via* the IRM may be beneficial to forecast immune responses and avert immunosuppressive therapy in sufferers, who do not respond immunologically.

HCC arises on the background of chronic liver disease. Despite the development of effective anti-viral therapeutics, HCC is continuing to rise. Thus, many patients present with advanced disease out with the criteria for transplant, resection or even locoregional therapy. For patients who are not candidates of curative treatments, locoregional therapies such as transarterial chemoembolization (TACE), transarterial radioembolization (TARE), and stereotactic body radiation (SBRT) can improve survival and quality of life. Sorafenib, a multi-kinase VEGF inhibitor, is the most widely used systemic chemotherapy approved as a first-line agent for unresectable or advanced HCC. Whilst checkpoint inhibitors are at the forefront of this revolution, other therapeutics such as inhibitory cytokine blockade, oncolytic viruses, adoptive cellular therapies and vaccines are emerging (72, 73). This study identified the potential sensitive drugs for patients in different IRG score groups, and the combination of these drugs and targeting angiogenesis may contribute to alleviating drug resistance and improving clinical outcomes. Furthermore, the effectiveness of immunotherapy requires specific biomarkers as a predictive pattern. TIDE and IPS signatures have been created to evaluate ICIs response. Accordingly, we observed that virus-related HCC patients with low IRG scores displayed low TIDE scores. All the above results demonstrate IRGs is an advantageously predictive tool in precision immunotherapy for virus-related HCC patients.

## 5 Conclusion

In conclusion, we have summarized the prognostic role of inflammation-related regulatory genes in virus-related HCC patients and then constructed a prognostic model based on IRGs involving six genes, which can accurately and stably predict survival and guide individualized treatment decisions in virus-related HCC patients. We further found that alterations in TME characteristics may be a potential mechanism of this model to predict the prognosis of virus-related HCC patients. Although we verified the stability of the risk model from multiple aspects, there are still some limitations. First, further studies with a large sample size are required to draw definitive conclusions. Furthermore, extensive prospective studies are necessary to gain insight into the relationship between risk scores and TME *in vivo* and vitro models.

## Data availability statement

The original contributions presented in the study are included in the article/**Supplementary Material**. Further inquiries can be directed to the corresponding author.

## Ethics statement

The studies involving human participants were reviewed and approved by the Ethical Committee of Chengdu Medical College approved the present study. Written informed consent was obtained from each participant by the institutional guidelines. The patients/participants provided their written informed consent to participate in this study. The animal study was reviewed and approved by the Ethical Committee of Chengdu Medical College approved the present study.

## Author contributions

YH designed the study, performed bioinformatics and experiments. Y-jG analyzed the data and prepared the figures. YH wrote the manuscript. S-rL revised the manuscript. All of the authors read and approved the final manuscript.

## Supplementary material

Supplementary Figure 1 Validation of prognostic models for six inflammation-related signatures. **(A–C)** risk score distribution, survival status, the expression level of hub genes, Principal component analysis, and Kaplan-Meier curves at different risk groups from ICGA database **(A)** and GSE84337 database **(B)**. *p < 0.05.

Supplementary Figure 2 The correlation analysis of IRG and clinicopathological variables and corresponding stratification analysis in virus-related HCCs. The correlation between IRG and **(A)** Age, **(B)** Alcohol consumption, **(C)** Gender, **(D)** TNM stage, **(E)** Fetoprotein, and **(F)** Radiation therapy and its corresponding OS analysis. *p < 0.05, **p < 0.01, ***p < 0.001.

Supplementary Figure 3 Correlations between IRG and immune cell types.

## References

[1] Tsai WL Chung RT. Viral hepatocarcinogenesis. Oncogene (2010) 29(16):2309–24. doi: 10.1038/onc.2010.36 PMC3148694 20228847

[2] Ringelhan M McKeating Jane A Protzer U. Viral hepatitis and liver cancer. Philos Trans R Soc Lond B Biol Sci (2017) 372(1732):20160274. doi: 10.1098/rstb.2016.0274 28893941 PMC5597741

[3] Loureiro D Tout I Narguet S. miRNAs as potential biomarkers for viral hepatitis b and c. Viruses (2020) 12(12):1440. doi: 10.3390/v12121440 33327640 PMC7765125

[4] Sadri Nahand J Bokharaei-Salim F Salmaninejad A Nesaei A Mohajeri F Moshtzan A. microRNAs: Key players in virus-associated hepatocellular carcinoma. J Cell Physiol (2019) 234(8):12188–225. doi: 10.1002/jcp.27956 PMC13484166 30536673

[5] Shlomai A Jong YD Rice CM. Virus associated malignancies: The role of viral hepatitis in hepatocellular carcinoma. Semin Cancer Biol (2014) 26:78–88. doi: 10.1016/j.semcancer.2014.01.004 24457013 PMC4048791

[6] Lee J Tsai K-N James OuJ-H. Mechanisms of hepatitis b virus-induced hepatocarcinogenesis. Recent Results Cancer Res (2021) 217:47–70. doi: 10.1007/978-3-030-57362-1_3 33200361

[7] Stanislas P Sylvie. The remarkable history of the hepatitis c virus. Genes Immun (2019) 20(5):436–46. doi: 10.1038/s41435-019-0066-z 31019253

[8] Okusaka T Ikeda M Morizane C. Chemotherapy for hepatocellular carcinoma: Current status and future perspectives. Jpn J Clin Oncol (2018) 48(2):103–14. doi: 10.1093/jjco/hyx180 29253194

[9] Zhao Y Zhang YN Wang KT. Lenvatinib for hepatocellular carcinoma: From preclinical mechanisms to anti-cancer therapy. Biochim Biophys Acta (BBA) - Rev Cancer (2020) 1874(1):188391. doi: 10.1016/j.bbcan.2020.188391 32659252

[10] Demir T Lee SS Kaseb AO. Systemic therapy of liver cancer. Adv Cancer Res (2021) 149:257–94. doi: 10.1016/bs.acr.2020.12.001 33579425

[11] PJohnston M Khakoo S. Immunotherapy for hepatocellular carcinoma: Current and future. World J Gastroenterol (2019) 25(24):2977–89. doi: 10.3748/wjg.v25.i24.2977 PMC6603808 31293335

[12] Keenan BP Fong L Kelley RK. Immunotherapy in hepatocellular carcinoma: the complex interface between inflammation, fibrosis, and the immune response. J Immunotherapy Cancer (2019) 7(1):267. doi: 10.1186/s40425-019-0749-z PMC6798343 31627733

[13] Oura K Morishita A Tani J Masaki T. Tumor immune microenvironment and immunosuppressive therapy in hepatocellular carcinoma: A review. Int J Mol Sci (2021) 22(11):5801. doi: 10.3390/ijms22115801 34071550 PMC8198390

[14] Nakatsu G. Toward a postbiotic era of microbiome science: Opportunities to advance immunotherapies for hepatocellular carcinoma. J Gastroenterol Hepatol (2022) 37(1):34–8. doi: 10.1111/jgh.15715 34665475

[15] Woller N Kühnel F. Virus infection, inflammation and prevention of cancer. Rev Recent Results Cancer Res (2014) 193:33–58. doi: 10.1007/978-3-642-38965-8_3 24008292

[16] Read SA Douglas MW. Virus induced inflammation and cancer development. Cancer Lett (2014) 345(2):174–81. doi: 10.1016/j.canlet.2013.07.030 23941825

[17] J m C Chew V. Impact of viral etiologies on the development of novel immunotherapy for hepatocellular carcinoma. Semin Liver Dis (2019) 40(2):131–42. doi: 10.1055/s-0039-3399534 31810095

[18] Yang Y Kim S Seki E. Inflammation and liver cancer: Molecular mechanisms and therapeutic targets. Semin Liver Dis (2019) 39(1):26–42. doi: 10.1055/s-0038-1676806 30809789 PMC6616367

[19] Marc R Dominik P Tracy O. The immunology of hepatocellular carcinoma. Nat Immunol (2018) 19(3):222–32. doi: 10.1038/s41590-018-0044-z 29379119

[20] Demaria S ikarsky E Karin M Eli P Mathias H. Cancer and inflammation: Promise for biologic therapy. J Immunotherapy (2010) 3(4):335. doi: 10.1097/CJI.0b013e3181d32e74 PMC2941912 20386472

[21] Mathai AM Alexander J Kuo FY Torbenson M Swanson PE Yeh MM. Type II ground-glass hepatocytes as a marker of hepatocellular carcinoma in chronic hepatitis b. Hum Pathol (2013) 44(8):1665–71. doi: 10.1016/j.humpath.2013.01.020 23574780

[22] Prieto J. Inflammation, HCC and sex: IL-6 in the centre of the triangle. J Hepatol (2008) 48(2):380–1. doi: 10.1016/j.jhep.2007.11.007 18093689

[23] Kumar RA Biju P Balasubramaniyan V. Increased systemic zonula occludens 1 associated with inflammation and independent biomarker in patients with hepatocellular carcinoma. BMC Cancer (2018) 18(1):572–. doi: 10.1186/s12885-018-4484-5 PMC5960107 29776350

[24] Reghupaty SC Mendoza R Sarkar D. AEG-1 targeting for inhibiting inflammation: potential anti-HCC strategy. Oncotarget (2019) 10(6):629–30. doi: 10.18632/oncotarget.26602 PMC6363015 30774759

[25] Esparza-Baquer A Labiano I Sharif O Oakley F Hijona E Jimenez-Aguero R. The anti-inflammatory receptor TREM2 halts the generation of HCC in mice through the inhibition of liver inflammation and hepatocyte proliferative responses. J Hepatol (2018) 68:S675–6. doi: 10.1016/S0168-8278(18)31610-6

[26] Yu S Wang Y Jing L. Autophagy in the "inflammation-carcinogenesis" pathway of liver and HCC immunotherapy. Cancer Lett (2017) 411:82–9. doi: 10.1016/j.canlet.2017.09.049 28987386

[27] Miao L Zhang Z Ren Z. Application of immunotherapy in hepatocellular carcinoma. Front Oncol (2021) 11:699060. doi: 10.3389/fonc.2021.699060 34513678 PMC8426571

[28] Linderman GC Steinerberger S. Clustering with t-SNE. J Mach Learn Res (2019) 1(2):313–32. doi: 10.1137/18M1216134 PMC7561036 33073204

[29] Monti S Tamayo P Mesirov JP Golub TR. Consensus clustering: A resampling-based method for class discovery and visualization of gene expression microarray data. (2003) 52(1-2):91–118. doi: 10.1023/A:1023949509487

[30] Meng Z Ren D Zhang K Wu H. Using ESTIMATE algorithm to establish an 8-mRNA signature prognosis prediction system and identify immunocyte infiltration-related genes in pancreatic adenocarcinoma. Aging (2020) 12(6):5048–70. doi: 10.18632/aging.102931 PMC7138590 32181755

[31] Wang Y Hou H Lin C Kuo Y Yao C Hsu C. A CIBERSORTx-based immune cell scoring system could independently predict the prognosis of patients with myelodysplastic syndromes. Blood Adv (2021) 5(22):4535–48. doi: 10.1182/bloodadvances.2021005141 PMC8759137 34614508

[32] Hu X Shen F Zhao Z Qu X Ye J. An individualized gait pattern prediction model based on the least absolute shrinkage and selection operator regression. J Biomechanics (2020) 112(16):110052. doi: 10.1016/j.jbiomech.2020.110052 33039924

[33] Hui HQ Ding LX University W. Rapid robust clustering algorithm for Gaussian finite mixture model. Comput Sci (2013). doi: 10.1016/j.patcog.2006.09.012

[34] Vickers AJ Elkin EB. Decision curve analysis: A novel method for evaluating prediction models. Med Decision Making (2006) 26(6):565–74. doi: 10.1177/0272989X06295361 PMC2577036 17099194

[35] Mayakonda A Lin DC Assenov Y Plass C Koeffler HP. Maftools: Efficient and comprehensive analysis of somatic variants in cancer. Genome Res (2018) 28(11). doi: 10.1101/gr.239244.118 PMC6211645 30341162

[36] Paul G Nancy C Huang RS. pRRophetic: An r package for prediction of clinical chemotherapeutic response from tumor gene expression levels. PLoS One (2014) 9(9):e107468–e107468. doi: 10.1371/journal.pone.0107468 25229481 PMC4167990

[37] Munir T. Microscopic-observation drug-susceptibility assay for the diagnosis of TB. New Engl J Med (2006) 355(15):1539. 17035648 10.1056/NEJMoa055524 PMC1780278

[38] Samstein RM Lee CH Shoushtari AN. Tumor mutational load predicts survival after immunotherapy across multiple cancer types. Nat Genet (2019) 51(2):202–6. doi: 10.1038/s41588-018-0312-8 PMC6365097 30643254

[39] Sahin IH Akce M Alese O. Immune checkpoint inhibitors for the treatment of MSI-H/MMR-D colorectal cancer and a perspective on resistance mechanisms. Br J Cancer (2019) 121(10):809–18. doi: 10.1038/s41416-019-0599-y PMC6889302 31607751

[40] Xu Q Xu H Chen S Zhu Y Huang W. Immunological value of prognostic signature based on cancer stem cell characteristics in hepatocellular carcinoma. Front Cell Dev Biol (2021) 9:710207. doi: 10.3389/fcell.2021.710207 34409040 PMC8365341

[41] Nishino K Umezawa A. Induced pluripotent stem cells from human extra-embryonic amnion cells: Role of DNA methylation in maintaining stemness. Springer Netherlands (2012) 4:59–65.

[42] Fu J Li K Zhang W. Large-Scale public data reuse to model immunotherapy response and resistance. Genome Med (2020) 12(1):21. doi: 10.1186/s13073-020-0721-z 32102694 PMC7045518

[43] Charoentong P Finotello F Angelova M Mayer C Efremova M Rieder D. Pan-cancer immunogenomic analyses reveal genotype-immunophenotype relationships and predictors of response to checkpoint blockade. Cell Rep (2017) 18(1):248–62. doi: 10.1016/j.celrep.2016.12.019 28052254

[44] Lampertico P Agarwal K Berg T. Clinical practice guidelines on the management of hepatitis b virus infection. J Hepatol (2017) 67(2):370–98. doi: 10.1016/j.jhep.2017.03.021 28427875

[45] Sarmati L Malagnino V. HBV infection in HIV-driven immune suppression. Viruses (2019) 11(11):1077. doi: 10.3390/v11111077 31752284 PMC6893694

[46] Tang W Chen Z Zhang W Cheng Y Wang X. The mechanisms of sorafenib resistance in hepatocellular carcinoma: Theoretical basis and therapeutic aspects. Signal Transduct Target Ther (2020) 5(1):87. 32532960 10.1038/s41392-020-0187-x PMC7292831

[47] Qin S Bi F Gu S. Donafenib versus sorafenib in first-line treatment of unresectable or metastatic hepatocellular carcinoma: A randomized, open-label, parallel-controlled phase II-III trial. J Clin Oncol (2021) 39(27):3002–11. doi: 10.1200/JCO.21.00163 PMC8445562 34185551

[48] Tsuchiya N Yu S Endo I Saito K Uemura Y Nakatsura T. Biomarkers for the early diagnosis of hepatocellular carcinoma. World J Gastroenterol (2015) 21(37):10573–83. doi: 10.3748/wjg.v21.i37.10573 PMC4588079 26457017

[49] Han J Han M-l Xing H. Tissue and serum metabolomic phenotyping for diagnosis and prognosis of hepatocellular carcinoma. Int J Cancer (2020) 146(6):1741–53. doi: 10.1002/ijc.32599 31361910

[50] Yu JI Park HC Yoo GS Paik SW Nam H. Clinical significance of systemic inflammation markers in newly diagnosed, previously untreated hepatocellular carcinoma. Cancers (2020) 12(5):1300. doi: 10.3390/cancers12051300 32455607 PMC7281027

[51] Lin W-W Karin M. Cytokine-mediated link between innate immunity, inflammation, and cancer. J Clin Invest (2007) 117(5):1175–83. doi: 10.1172/JCI31537 PMC1857251 17476347

[52] Ben-Baruch A. Inflammation-associated immune suppression in cancer: The roles played by cytokines, chemokines and additional mediators. Semin Cancer Biol (2006) 16(1):38–52. doi: 10.1016/j.semcancer.2005.07.006 16139507

[53] Tanaka T Narazaki M Kishimoto T. IL-6 in inflammation, immunity, and disease. Cold Spring Harbor Perspect Biol (2014) 6(10):a016295. doi: 10.1101/cshperspect.a016295 PMC4176007 25190079

[54] Guijarro C Egido J. Transcription factor-kappa b (NF-kappa b) and renal disease. Kidney Int (2001) 59(2):415–24. doi: 10.1046/j.1523-1755.2001.059002415.x 11168923

[55] Dinarello CA. Proinflammatory cytokines. Chest (2000) 118(2):503–8. doi: 10.1378/chest.118.2.503 10936147

[56] Ge W Hou C Zhang W Guo X Gao P Song X. Mep1a contributes to ang II-induced cardiac remodeling by promoting cardiac hypertrophy, fibrosis and inflammation. J Mol Cell Cardiol (2021) 152(5):52–68. doi: 10.1016/j.yjmcc.2020.11.015 33301800

[57] Wang XM Hamza M Wu TX Dionne RA. Upregulation of IL-6, IL-8 and CCL2 gene expression after acute inflammation: Correlation to clinical pain. Pain (2009) 142(3):275–83. doi: 10.1016/j.pain.2009.02.001 PMC3513699 19233564

[58] Ehrentraut H A Westrich J K Eltzschig H Clambey ET Tobias E. Adora2b adenosine receptor engagement enhances regulatory T cell abundance during endotoxin-induced pulmonary inflammation. PLoS One (2012) 7(2):e32416. doi: 10.1371/journal.pone.0032416 22389701 PMC3289657

[59] Yu LS Yu G Hai FG. TNFSF9 exerts an inhibitory effect on hepatocellular carcinoma. J Dig Dis (2017) 18(7):395–403. 28547807 10.1111/1751-2980.12489

[60] Miok K Seon-Jin L Sangsu S. Novel natural killer cell-mediated cancer immunotherapeutic activity of anisomycin against hepatocellular carcinoma cells. Sci Rep (2018) 8(1):10668–. doi: 10.1038/s41598-018-29048-8 PMC6045618 30006566

[61] Xia S Wu J Zhou W Zhang M Liao J. SLC7A2 deficiency promotes hepatocellular carcinoma progression by enhancing recruitment of myeloid-derived suppressors cells. Cell Death Disease.Cell Death Dis (2021) 12(6):570. doi: 10.1038/s41419-021-03853-y 34108444 PMC8190073

[62] Han-Yue O Jing X Luo J Ru‐Hai Zou Chen K Le Y. MEP1A contributes to tumor progression and predicts poor clinical outcome in human hepatocellular carcinoma. Hepatology (2016) 63(4):1227–39. doi: 10.1002/hep.28397 26660154

[63] Li XG Yao WB Yuan Y Chen P Li B Li J. Targeting of tumour-infiltrating macrophages *via* CCL2/CCR2 signalling as a therapeutic strategy against hepatocellular carcinoma. Gut (2017) 66(1):157–67. doi: 10.1136/gutjnl-2015-310514 26452628

[64] Yuan XY Mills TT Doursout MF Evans SE Vidal Melo MF Eltzschig HK. Alternative adenosine receptor activation: The netrin-Adora2b link. Front Pharmacol (2022) 13:944994. doi: 10.3389/fphar.2022.944994 35910389 PMC9334855

[65] Liao J Zeng DN Li JZ Hua QM Xiao Z He C. Targeting adenosinergic pathway enhances the anti-tumor efficacy of sorafenib in hepatocellular carcinoma. Hepatol Int (2020) 14(1):80–95. doi: 10.1007/s12072-019-10003-2 31802389

[66] Kay JE Thadhani E Samson LD. Inflammation-induced DNA damage, mutations and cancer. DNA Repair (2019) 83:102673. doi: 10.1016/j.dnarep.2019.102673 31387777 PMC6801086

[67] Huo J Wu L Zang Y. A prognostic model of 15 immune-related gene pairs associated with tumor mutation burden for hepatocellular carcinoma. Front Mol Biosci (2020) 7:581354. doi: 10.3389/fmolb.2020.581354 33282911 PMC7691640

[68] Grawitz PB. Inflammation and cancer. Nature (2015) 420(6917):860–7. 10.1038/nature01322 PMC2803035 12490959

[69] Greten FR Grivennikov SI. Inflammation and cancer: Triggers, mechanisms, and consequences. Immunity (2019) 51(1):27–41. doi: 10.1016/j.immuni.2019.06.025 31315034 PMC6831096

[70] Liu ZY Liu X Liang JX. Immunotherapy for hepatocellular carcinoma: Current status and future prospects. Front Immunol (2021) 12:765101. doi: 10.3389/fimmu.2021.765101 34675942 PMC8524467

[71] Zhuang W Zhang Z Zhang S. An immunogenomic signature for molecular classification in hepatocellular carcinoma. Mol Therapy-Nucleic Acids (2021) 25:105–15. doi: 10.1016/j.omtn.2021.06.024 PMC8332372 34401208

[72] Couri T Pillai A. Goals and targets for personalized therapy for HCC. Hepatol Int (2019) 13(2):125–37. doi: 10.1007/s12072-018-9919-1 30600478

[73] Foerster F Gairing SJ Müller L. NAFLD-driven HCC: Safety and efficacy of current and emerging treatment options. J Hepatol (2022) 76(2):446–57. doi: 10.1016/j.jhep.2021.09.007 34555422

