# Supplementary figures and images for: An inflammation-related gene landscape predicts prognosis and response to immunotherapy in virus-associated hepatocellular carcinoma
Source: Front Oncol. 2023 Mar 9;13:1118152. doi: 10.3389/fonc.2023.1118152 (PMC10033597; doi:10.3389/fonc.2023.1118152)

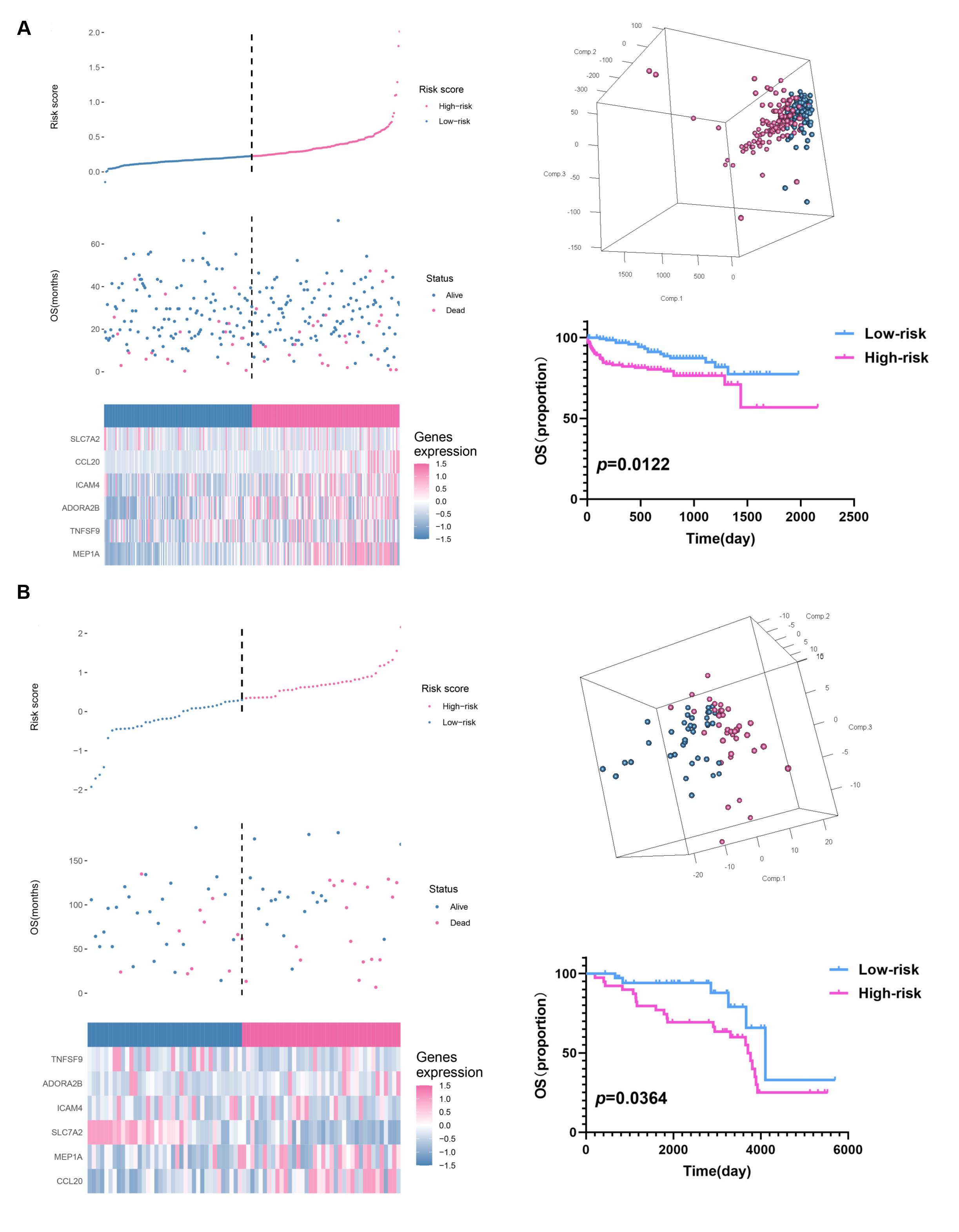

Supplement: Supplementary Figure 1 — Validation of prognostic models for six inflammation-related signatures. (A–C) risk score distribution, survival status, the expression level of hub genes, Principal component analysis, and Kaplan-Meier curves at different risk groups from ICGA database (A) and GSE84337 database (B). *p < 0.05. [file Image_1.jpeg]

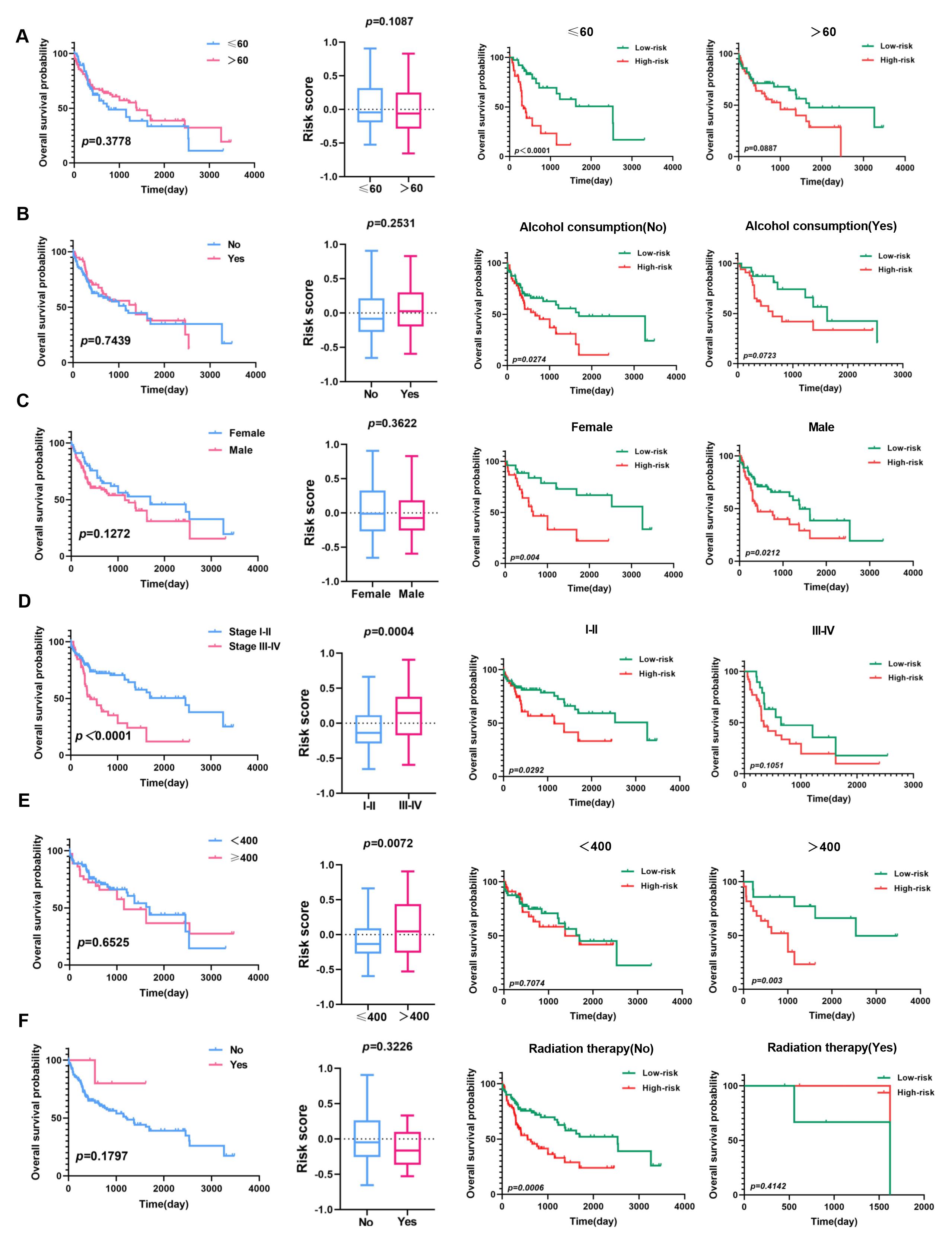

Supplement: Supplementary Figure 2 — The correlation analysis of IRG and clinicopathological variables and corresponding stratification analysis in virus-related HCCs. The correlation between IRG and (A) Age, (B) Alcohol consumption, (C) Gender, (D) TNM stage, (E) Fetoprotein, and (F) Radiation therapy and its corresponding OS analysis. *p < 0.05, **p < 0.01, ***p < 0.001. [file Image_2.jpeg]

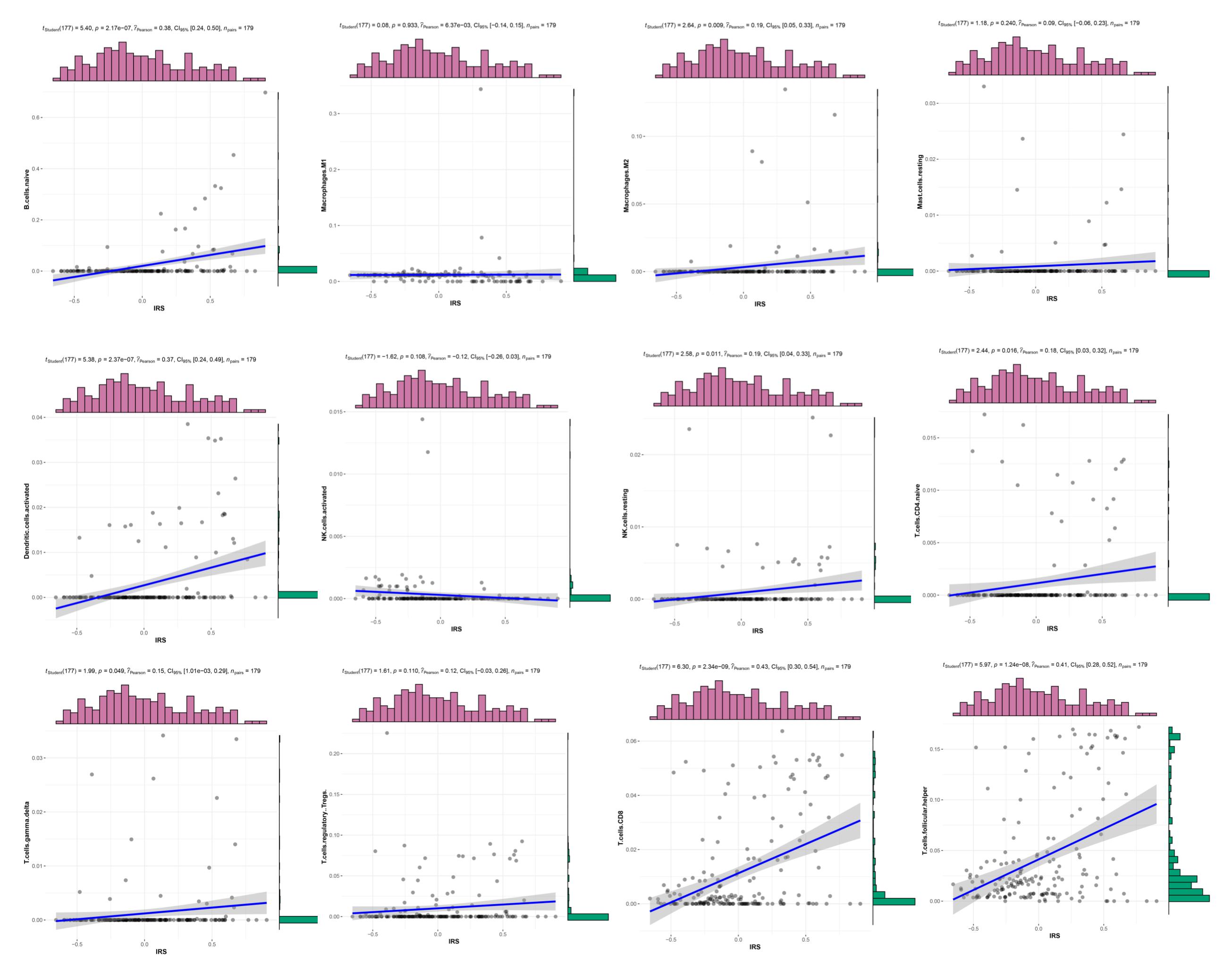

Supplement: Supplementary Figure 3 — Correlations between IRG and immune cell types. [file Image_3.jpeg]
